# The Association between Yang-Deficient Constitution and Clinical Outcome of Highly Active Antiretroviral Therapy on People Living with HIV

**DOI:** 10.1155/2013/201857

**Published:** 2013-12-30

**Authors:** Yuwen Cen, Ross Ka-kit Leung, Fuchun Zhang, Weidong Jia, Jiansheng Zhang, Xinghua Tan, Feilong Xu

**Affiliations:** ^1^Department of Infectious Diseases, Guangzhou 8th People's Hospital, 627 Dongfeng Dong Road, Guangzhou 510060, China; ^2^Stanley Ho Centre for Emerging Infectious Diseases, The Chinese University of Hong Kong, Hong Kong

## Abstract

*Objective*. To determine the association between Yang-Deficient Constitution and the clinical outcomes of HIV/AIDS patients who have initiated highly active antiretroviral therapy (HAART). *Method*. A total of 197 antiretroviral-naive adults who initiated HAART between 2009 and 2011 were recruited. The participants were asked to complete a questionnaire twice to assess their Yang-Deficient Constitution status before HAART. During the study, signs and symptoms and CD4 or CD8 T cell counts were recorded. Routine blood and biochemical tests were conducted. For the patients who were found to have infections, pathologic examination was performed. Statistical test of association of clinical attributes and demographic factors with Yang-Deficient Constitution was conducted. *Result*. Good test-retest reliability was observed for Yang-Deficient Constitution scoring. The median Yang-Deficient Constitution score of 142 eligible participants was 25. Female (score = 32.14, *P* < 0.05), hepatotoxicity (32.14, *P* < 0.1), nephrotoxicity (37.50, *P* < 0.1), total number of adverse events (*P* < 0.1), and mortality (39.29, *P* < 0.05) were associated with Yang-Deficient Consitution, while annual changes or nadir values of CD4 or CD8 T lymphocytes, and newly acquired infections after starting HAART were not. Mortality was also associated with total number of adverse events (*P* < 0.05), hepatotoxicity (*P* < 0.05), and nephrotoxicity (*P* < 0.05). *Conclusion*. Yang-Deficient Constitution score has a potential to be developed as a predictor for early HIV-related mortality and side effects. The interrelation and underlying mechanisms should be further investigated for evidence-based design of a more appropriate treatment strategy.

## 1. Introduction

After China's National Free Antiretroviral Treatment Program has been initiated since 2002, the rate of receiving antiretroviral therapy cases among the eligible HIV infection reached 84% nowadays. Although the mortality rate has decreased to 50% (*n* = 17740) for the 34,157 new infections of HIV, according to the 2012 report by the National Health and Family Planning Commission of the People's Republic of China, AIDS is still the top death-causing disease. Many of the newly admitted cases of HIV infections are indeed in the end stage of AIDS. Poor immunological response and adverse events are also common during initial highly active antiretroviral therapy (HAART). These partially account for early death in the first year of HAART [1].

Individual differences are known to have important impact on the progression of diseases. In particular, differences in the composition of genome account for many of the variations observed and more evidence is emerging that much of the wisdom of traditional medicine can in fact be explained at genomics level (Joshi et al. [2] and the references therein). Recently, HLA class II polymorphisms were found associated with the physiologic characteristics defined by Traditional Chinese Medicine (TCM) [3]. In TCM, constitution is believed to be a distinct characteristic of an individual [4]. Constitution has been used to guide disease prevention, health care, and medical practice [5]. Yang-Deficient Constitution is one of the major constitutions, which is characterized by chills, cold limbs, and also spontaneous sweating; loose tools and/or profuse clear urine; and lassitude. Recently, the molecular mechanisms of Yang-Deficient Constitution [6] have been studied, bridging the gap between conventional wisdom and knowledge gained from modern scientific advance. Different types of constitution may be related to specific lifestyle or diet habits [7] and manifest corresponding psychological characteristics of personality [8]. People of balanced constitution have better quality of life than those of imbalanced constitution [9]. Moreover, imbalanced constitution seemed to have strong correlation with chronic diseases such as obesity [10] and hypertension [11].

Since the last few years before the initiation of this study, we have been observing that HIV-infected patients of Yang-Deficient constitution are more likely to die in spite of receiving HAART. Yang deficiency Syndrome indeed appeared in many emergency and serious diseases, including the late stage of AIDS. Processed aconite root (a Chinese medicine) was shown to prevent cold-stress-induced hypothermia and immunosuppression in mice [12]. Higenamine and its enantiomer, the active ingredients of aconite root, reduced iNOS expression and NO production, suppressed inflammatory reactions, and increased survival rates in LPS-treated mice [13, 14]. Herbal composite formulae injection with aconite being the active ingredient was proved to protect the important organs during emergency and serious diseases [15–17]. We also found that aconite appears to alleviate the symptoms complained by our patients. We hypothesize that Yang-Deficient Constitution is associated with the prognosis of AIDS. To systematically investigate whether there is any association between Yang-Deficient Constitution and complications incurred from HIV infection and design evidence-based treatment, we conducted a prospective observational study for evaluating the association between Yang-Deficient Constitution and clinical outcomes.

## 2. Method

### 2.1. Study Population and Design

Treatment-naive HIV-1 infected patients aged 18 years or older at the Guangzhou 8th People's Hospital between January 1, 2010, and May 1, 2012, who joined the National Free Antiretroviral Treatment Program with a combination of at least three drugs, including NRTIs, PIs, and NNRTIs, were enrolled. Subjects were excluded if they were pregnant or unable to understand or finish the questionnaire due to linguistic or mental problems. All subjects received a full explanation of the study and provided written informed consent. The study was approved by ethics committees of Guangzhou 8th People's Hospital. The recruited patients were asked to complete the constitution questionnaire before the initiation of HAART. They were then scheduled follow-up visits once every 3 months. This study period was one year and the dropouts were also excluded.

We adopted the definitions published by Centers for Disease Control and Prevention revision of the AIDS in 1993 case definition (1993 Revised Classification System for HIV Infection and Expanded Surveillance Case Definition for AIDS Among Adolescents and Adults, http://www.cdc.gov/mmwr/preview/mmwrhtml/00018179.htm). People without an AIDS defining disease but with a CD4 cell count below 200 cells/L were classified as having AIDS.

We adopted the Standards of Classification and Determination of Constitution of Chinese Medicine issued by the China Association of Traditional Chinese Medicine in 2009, which includes the Classification and Determination of Yang-Deficient Constitution Scale (see Table 1). The original standardized questionnaire had been published in 2006 [18], which was then evaluated and proved to be effective in assessment on the constitution of healthy people and patients [19, 20]. Table 1 shows the English version translated by our group. During the interview, the interviewees were given a Chinese version to complete. The patients were also requested to complete this questionnaire again after the first interview to assess the reliability of their response. Raw sum scores were calculated for the constitution, and then the sum scores were converted to a conversion score on a 0-to-100 scale.

### 2.2. Sociodemographics, Drug Regimen, and Clinical Outcome Measures

We investigated gender, age, Yang-Deficient Constitution score, survival, HIV disease staging according to the definition by CDC, Highly Active Antiretroviral Therapy Medicine (3TC, AZT, D4T, EFV, NVP, KALETRA, and TDF), CD4 and CD8 count at baseline and 3, 6, 9, and 12 months after HAART, infections acquired before and after HAART (hepatitis B, hepatitis C, pulmonary tuberculosis, extrapulmonary tuberculosis, pneumonia, superficial fungal infections, visceral fungal infections, intestinal infections, encephalitis and meningitis, and sexually transmitted disease), immune reconstitution inflammatory syndrome, the number of days after HAART initiation for an infection to occur, adverse events including fatigue, allergy, alopecia, gastrointestinal reaction, mouth epithelium ulcer and pharyngitis, cerebral symptom, arthralgia, diabetes, hematologic toxicity, hepatotoxicity, abnormal lipid metabolism, pancreatitis, nephrotoxicity, sensory nerve dysfunction, and hyperlactacidemia, and total number of adverse events. The routine blood and biochemical tests conducted and their normal range are shown in Table 2. Attributes that had fewer than 10% of unique values relative to the number of samples and the ratio of the frequency of the most common value to the frequency of the second most common value larger than (95 : 5) were regarded as low variance attributes and discarded unless otherwise specified.

### 2.3. Data Management and Analysis

Reliability of duplicate tests was evaluated. To assure quality, patients were requested to complete the same questionnaire twice in two visits (separated by one week) and the absolute differences of the scores between the two visits were calculated. Local outlier factor (LOF), an algorithm for identifying density-based local outliers [21], was used to filter the unreliable data. Data errors and problems were identified and sent to clinical doctors for review. The remaining subjects then had CD4 and CD8 count and other relevant clinical information recorded. Sixteen clusters were used to classify the patterns in CD4 and CD8 reconstitution to represent increase or decrease for the transition between 0, 3, 6, 9, and 12 months after the initiation of HAART. Statistical analyses and self-organizing map were done by R (version 2.15.2).

## 3. Result

We recruited 197 HIV/AIDS patients but only 158 of whom completed the questionnaire twice. There was no difference between test-retest scores for two-third of the cases and about 90% with 1 or fewer (Q1: 147, Q2: 143, Q3: 138, Q4: 137, Q5: 146, Q6: 143, and Q7: 145) absolute score difference (Table 3). Nine of these subjects having total absolute score difference larger than 8 (9, 10, 11, 13, and 16) were outliners, defined by local outlier factor. These individuals were thus excluded from further investigation.

The other 149 patients were assigned scores by taking average of two visits, along with 39 patients that only completed once. As a result, there were 188 HIV/AIDS subjects at the beginning. Some of them dropped out during followups and finally there were 142 HIV positive individuals for in-depth analysis, with 57 (40%), 16 (11%), and 69 (49%) of whom were classified as types A, B, and C, respectively, according to CDC 1993 Revised Classification System (1993 Revised Classification System for HIV Infection and Expanded Surveillance Case Definition for AIDS Among Adolescents and Adults). The details of demographical information are summarized in Table 4.

HAART drugs 3TC, KALETRA, TDF, encephalitis and meningitis, previous diagnosis of sexually transmitted disease, newly acquired hepatitis B, hepatitis C, pulmonary and extrapulmonary TB, pneumonia, superficial and visceral fungal infections, alopecia, mouth epithelium ulcer and pharyngitis, arthralgia, diabetes, and pancreatitis were excluded for further analyses due to their low variance. Other attributes that were not significantly associated (*P* > 0.1) with Yang deficiency were also excluded.

**Figure 1:**
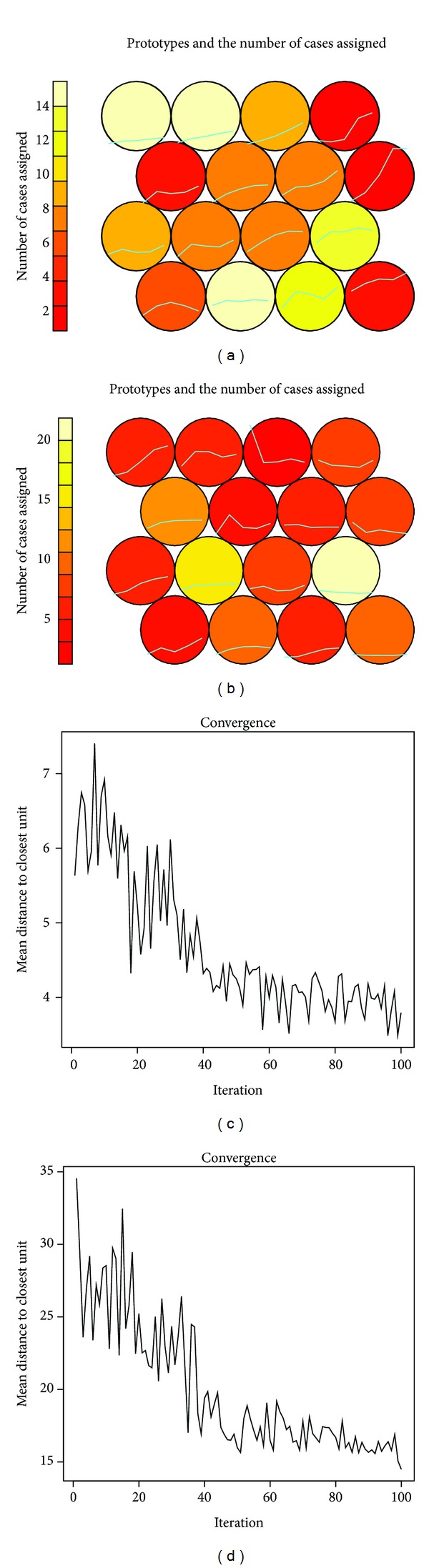
Change of CD4 and CD8 count over a one-year period during receiving HAART. ((a), (b)) units for the scale prototypic CD4 (a) and CD8 (b) change and the number of cases assigned. ((c), (d)) Convergence was attained at about the 50th iteration for both CD4 and CD8 profile analyses.

Compared to males females showed significantly more serious Yang deficiency (Table 5(a)), but age was not correlated with Yang deficiency. Yang deficiency was also associated with hepatotoxicity, nephrotoxicity, and the total number of side effects. Yang deficiency was neither associated with annual change nor Nadir value of CD4 or CD8 count. We also performed profile analysis for CD4+ and CD8+ T cell reconstitution by self-organizing map (Figure 1). Association was neither identified in CD4 (Kruskal-Wallis rank sum test *P* = 0.54) nor CD8 (Kruskal-Wallis rank sum test *P* = 0.34) reconstitution pattern (all the 16 clusters) with Yang deficiency. Yang deficiency was associated with both HIV disease staging and mortality.

There were 5 deaths in this study, due to pneumonia, infectious diarrhea, extrapulmonary tuberculosis, meningitis, encephalitis, and multiple organ dysfunction syndrome. The mortality rate was 3.5% (95% CI: 1.4%–8.6%). Indeed, the total number of newly acquired infections after starting HAART, previous pneumonia, intestinal infections, and IRIS and total number of the adverse events, particularly syndromes of hepatotoxicity and nephrotoxicity, were associated with mortality (Table 5(b)).

## 4. Discussion

Since the introduction of HAART in 2002 AIDS mortality (with and without receiving HAART) has begun to decrease from 39.2 to 14.2 per 100 person-years in 2009 in China [22]. In this study, the mortality rate during the first 12 months after HAART initiation was 3.5 deaths per 100 person-years, similar to the national statistics of 4.9 [1]. The causes of death in our patients were all AIDS-related diseases, similar to those reported in low- and middle-income countries [23].

In resource-limited settings, prioritization allows better allocation of resources for diagnostic tests and appropriate and timely health services. AIDS patients with low CD4+ cell counts incur higher expenditures in the first year of antiretroviral treatment, due to high incidence of adverse drug events and opportunistic infections. Annual costs of AIDS patients with opportunistic infections were 10 times higher than those of HIV infection alone [24], hardly adequately covered by public funding. Moreover, over half of the HIV-infected individuals live in the rural areas of China, according to the 2011 estimates for the HIV/AIDS epidemic in China by National Center for AIDS/STD Control and Prevention, China CDC. Public funding accounts for over 90% of all HIV/AIDS expenditures in the rural areas [25]. In rural settings, where trained personnel, facilities, and budget are all limiting, a self-report questionnaire is a possible option. Public health practitioners collect information usually by various ways and examine patients by signs and symptoms for diagnosis in order to design appropriate course of treatment and medications. More than 80% of respondents successfully completed the questionnaire twice in this study. Biomarkers have been extensively investigated as predictors for HIV clinical disease progression (AIDS or death) over the last two decades [26]. Nevertheless, due to equipment and technical limitations, the use of these biomarkers was no more efficient than CD4 cell count. Nevertheless, baseline CD4 cell count was a necessary but not sufficient condition for mortality. Poor CD4 cell reconstitution also did not definitely lead to death (Figure 1; two left topmost patterns). In this context, convenient and noninvasive, questionnaire-derived Yang-Deficient Constitution score has the potential to be developed as an independent or accessory predictor for disease progression or clinical outcomes. Larger-scale validation studies can be conducted to test its applicability.

Constitution is largely inborn and Yang deficiency is one of the manifestations. Independent of age, female patients had higher Yang-Deficient Constitution score, which alludes to possible genetic effects. Our result of the association of female with Yang-Deficient Consitution was consistent with the Yin-Yang theory in TCM, which says male is characterized by Yang while female Yin. With regard to age, according to Yellow Emperor's Canon of Traditional Chinese Medicine, Yang-qi changes along with age, but not in a monotonic manner. Most of the people recruited in our study were between 20 and 55 years old, change of Yang-qi during this age group may follow a bell-shaped pattern, thus Yang-Deficient Consitution was proved to have no linear correlation with age in this study. Whether the correlation between Yang-Deficient Consitution and gender was due to sex hormonal level needs further elucidation [27, 28]. Malnutrition is also a factor to be considered. Through a series of statistical tests, we have identified that Yang-Deficient Constitution was not associated with infection acquired after starting HAART, low baseline CD4 count, or CD4 cell reconstitution. However, Yang-Deficient Constitution was associated with death. It should be noted that, however, death was also associated with infections acquired after starting HAART. Since Yang-Deficient Constitution did not appear to predispose the patient to infections, they are likely to be independent risk factors leading to mortality. In our study, although HIV disease stage B patients were relatively Yang deficient in general, HIV disease staging was not the sufficient condition for mortality. Other concurrent conditions might have to satisfy, which ultimately lead to death.

Hepatotoxicity and nephrotoxicity might be the indirect causes of death of the AIDS patients, which are generally caused by overlapping anti-infection treatment during opportunistic infections. Liver injury can be resulted from antiretroviral treatment [29], including nonnucleoside reverse transcriptase inhibitors, which has been widely used by China's National Free Antiretroviral Treatment Program. Antituberculosis drug administration is another common cause of hepatotoxicity. TB is common co-infection with HIV in China [30] and a national campaign has already been implemented since 2010 for the prevention and control of tuberculosis and HIV coinfection. Counter attempts such as administration of anti-TB drugs are, however, hampered by hepatotoxicity induced during drug delivery [31]. In our study, Yang-Deficient Constitution appeared to increase the risk of antiretroviral or anti-TB drug induced hepatotoxicity. Nephropathy is another common complication of HIV-infected individuals. It is also associated with cardiovascular disease and mortality [32]. During the ART-era, HIV [32] and long-term HAART exposure [33] could be mixed causes for nephropathy. In our study, antiretroviral agents did not seem to be the reasons of nephrotoxicity, because indinavir, atazanavir, and tenofovir disoproxil fumarate (TDF), which have well-established associations with direct nephrotoxicity supported by numerous, consistent case reports and large cohort studies [33], were not used. However, the anti-infection antibiotics such as foscarnet sodium and amphotericin B may be the causes during cytomegaloviral or fungal infection.

**Figure 2:**
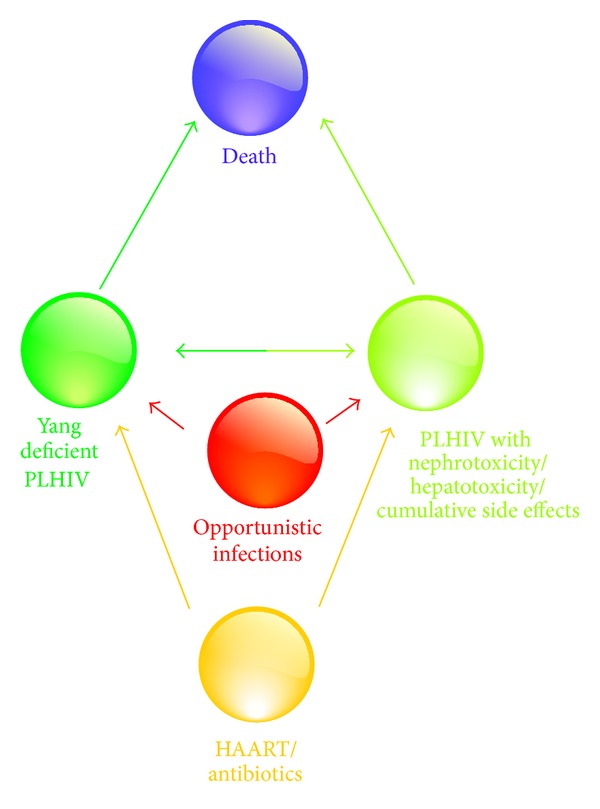
A hypothesis derived from the key findings about Yang-Deficient Constitution and clinical outcomes of AIDS patients. PLHIV: people living with HIV; HAART: highly active antiretroviral therapy.

To the best of our knowledge, this is the first report on the correlation between Yang-Deficient Constitution and hepatotoxicity and nephrotoxicity. Mitochondrial toxicity, commonly observed in HAART, might be responsible for hepatotoxicity and nephrotoxicity [34]. Mitochondrial dynamics indeed plays a key role in energy conversion and metabolism, cell life and death, and disease [35], and various aspects of metabolism, including lipid, amino acid, and energy metabolism, were imbalanced or weakened in Yang deficiency syndrome patients [36]. Whether Yang-Deficient Consitution is susceptible to mitochondrial toxicity is worth further investigation. A schematic diagram is shown in Figure 2 to summarize the key finding in this study. Females are prone to Yang deficiency. HIV-infected patients (or people living with HIV, PLHIV) with a Yang-Deficient Consitution, when administered HAART or antibiotics to control HIV replication and treat infections, may more easily lead to multiple side effects including nephrotoxicity and hepatotoxicity. When they also succumb to opportunistic infections, they may eventually lead to death. It is not known whether HAART/antibiotics or opportunistic infections aggravate PLHIV inflicted by toxic side effects and in turn lead to death.

## Figures and Tables

**Table 1:** The measuring scale for Yang-Deficient Consitution of Traditional Chinese Medicine.

Experience/condition in the past year	Never	Sometimes	Often	Usually	Always
(1) Do you feel cold at your limbs?	1	2	3	4	5
(2) Are you sensitive to cold at stomach, back, waist, or laps?	1	2	3	4	5
(3) Are you usually sensitive to cold weather and dress more than others?	1	2	3	4	5
(4) Are you more sensitive to cold air (such as outside of winter, air-conditioning, or electric fan)?	1	2	3	4	5
(5) Do you have flu- or cold-like symptoms more frequently or easily than others?	1	2	3	4	5
(6) Do you feel uncomfortable or worried for cold water or foods?	1	2	3	4	5
(7) Do you have pulpy bowel after having cold water or foods?	1	2	3	4	5

**Table 2:** Routine blood and biochemical tests conducted and their normal range.

Index	Normal range	Unit
Leukocyte	4–10	10*E*9/L
Neutrophils	2–7.5	10*E*9/L
Hemoglobin	120–160	g/L
Platelet	100–300	10*E*9/L
Seralbumin	35–55	g/L
Alkaline phosphatase	40–150	U/L
Total bilirubin	5.1–22.2	*μ*mol/L
Aspartate transaminase	5–40	U/L
Glutamic-oxaloacetic transaminase	5–40	U/L
Glucose	3.9–6.1	mmol/L
Cholesterol	3.1–6.0	mmol/L
Low density lipoprotein cholesterol	0–3.36	mmol/L
Triglyceride	0.79–1.70	mmol/L
Sarcosine kinase	26–174	U/L
Creatinine	44–133	*μ*mol/L
Pancreatic amylase	22–210	U/L
Lactate	0.6–2.2	mmol/L

**Table:** (a)

Absolute raw score difference	Frequency						
	Q1	Q2	Q3	Q4	Q5	Q6	Q7
0	115	105	118	100	107	116	107
1	32	38	20	37	39	27	38
2	9	15	14	17	10	11	11
3	2		6	2	2	3	2
4				2		1	

**Table:** (b)

Total absolute raw score difference	Frequency
0	61
1	11
2	14
3	11
4	12
5	15
6	12
7	7
8	6
9	1
10	2
11	3
13	2
16	1

**Table 4:** Characteristics of 142 HIV patients.

Category	Frequency/median
Gender	
Male	96
Female	46
Age	35
Yang-Deficient Constitution score	25
HIV disease staging	
A	57
B	16
C	69
Mortality	5
CD4 count	
Baseline	113
3 months after HAART	202
6 months after HAART	206
9 months after HAART	229
12 months after HAART	269
CD8 count	
Baseline	678.5
3 months after HAART	837
6 months after HAART	836
9 months after HAART	861
12 months after HAART	934
Highly active antiretroviral therapy	
3TC	142
AZT	45
D4T	95
EFV	71
NVP	62
KALETRA	7
TDF	1
Infection status before starting HAART	
Presence of infection before starting HAART	50
Hepatitis B	16
Hepatitis C	19
Pulmonary tuberculosis	30
Extrapulmonary tuberculosis	12
Pneumonia	42
Superficial fungal infections	37
Visceral fungal infections	31
Intestinal infections	10
Encephalitis and meningitis	5
Sexually transmitted disease	2
Newly acquired infection after HAART	
Presence of newly acquired infection after HAART	29
Pulmonary tuberculosis	1
Extrapulmonary tuberculosis	6
Pneumonia	7
Superficial fungal infections	2
Visceral fungal infections	6
Intestinal infections	8
Encephalitis and meningitis	2
Other infections	5
Immune reconstitution inflammatory syndrome (IRIS)	9
Adverse events	
Fatigue	18
Allergy	50
Alopecia	1
Gastrointestinal reaction	44
Mouth epithelium ulcer and pharyngitis	7
Cerebral symptom	26
Arthralgia	3
Diabetes	1
Hematologic toxicity	48
Hepatotoxicity	49
Abnormal lipid metabolism	26
Pancreatitis	0
Nephrotoxicity	11
Sensory nerve dysfunction	17
Hyperlactacidemia	18
Total number of side effects	24
Number of days an infection occurred after HAART initiation	80

**Table:** (a)

Variable	Category	Number/IQR	Correlation coefficient	Yang deficiency median score	*P* value*
Gender	Male	96	N/A	22.32	<0.05
	Female	46		32.14	
Hepatotoxicity	Yes	4 9	N/A	32.14	<0.1
	No	93		25.00	
Nephrotoxicity	Yes	11	N/A	37.50	<0.1
	No	131		25.00	
HIV disease staging	A	57	N/A	23.21	<0.05
	B	16		34.82	
	C	69		25.00	
Mortality	Yes	137	N/A	39.29	<0.05
	No	5		25.00	
Age	N/A	29–41	−0.028	ND	0.74
CD4	Annual change	77–217	−0.028	ND	0.75
	Nadir value	19–194	−0.053	ND	0.53
CD8	Annual change	−138–510	−0.028	ND	0.74
	Nadir value	334–737	−0.13	ND	0.12
Total number of adverse events	N/A	1–5	0.16	ND	<0.1

*Wilcoxon rank sum test with continuity correction, Spearman's rank correlation, and Kruskal-Wallis rank sum test were performed where appropriate.

**Table:** (b)

Variable		Alive	Dead	*P*	OR	OR 95% CI
Previous pneumonia	No	99	1	<0.05	10.42	1.48, 207.38
	Yes	38	4			
Intestinal infections	No	131	3	<0.05	14.55	1.69, 106.00
	Yes	6	2			
IRIS	No	130	3	<0.05	12.38	1.46, 87.72
	Yes	7	2			
Total number of newly acquired infections after HAART	N/A	N/A	N/A	<0.05	1.63	1.20, 2.41
Hepatotoxicity	No	92	1	<0.05	8.18	1.17, 162.48
	Yes	45	4			
Nephrotoxicity	No	129	2	<0.05	24.19	3.55, 205.36
	Yes	8	3			
Total number of adverse events	N/A	N/A	N/A	<0.05	7.85	1.35, 149.63
